# Bilateral Sensorineural Hearing Loss and Polyneuropathy in a Patient with Sweet's Syndrome

**DOI:** 10.1155/2015/751538

**Published:** 2015-12-06

**Authors:** Cather M. Cala, Lauren Kole, Naveed Sami

**Affiliations:** ^1^Tulane University, New Orleans, LA, USA; ^2^Yale University, New Haven, CT, USA; ^3^Department of Dermatology, University of Alabama-Birmingham, EFH 414, 1530 3rd Avenue S., Birmingham, AL 35294, USA

## Abstract

Sweet's syndrome is an inflammatory systemic disease which has been associated with various underlying causes. The disease can involve multiple areas of the body including the skin and neurological system. There have been only two cases which have described otological involvement. This report presents a patient who developed loss of hearing secondary to Sweet's syndrome after developing cutaneous involvement along with peripheral neuropathy. Despite the patient's skin and neuropathy noticing improvement with intravenous immunoglobulin and azathioprine, he required bilateral cochlear implants for partial recovery of his hearing loss. This case highlights the need to recognize Sweet's syndrome as a complicated disease process where the role of otolaryngologists is important in the multidisciplinary coordination of care in both diagnosis and treatment.

## 1. Introduction

Sweet's syndrome (SS), also known as “acute febrile neutrophilic dermatosis,” is an inflammatory systemic condition first described by Dr. Sweet in 1964 [1–3]. This rare disorder has been characterized by specific criteria [1, 2]. SS is often idiopathic but has been associated with systemic causes including an underlying malignancy, inflammatory disease, or infection. SS has been reported to involve other organ systems including neurological presentations. There have been only two reports in the English literature of bilateral sensorineural hearing loss (SNHL) associated with SS with no other neurological symptoms [4, 5]. In this report, we present a patient with Sweet's syndrome with both otological involvement and neurological involvement.

## 2. Case Report

**Figure 1:**
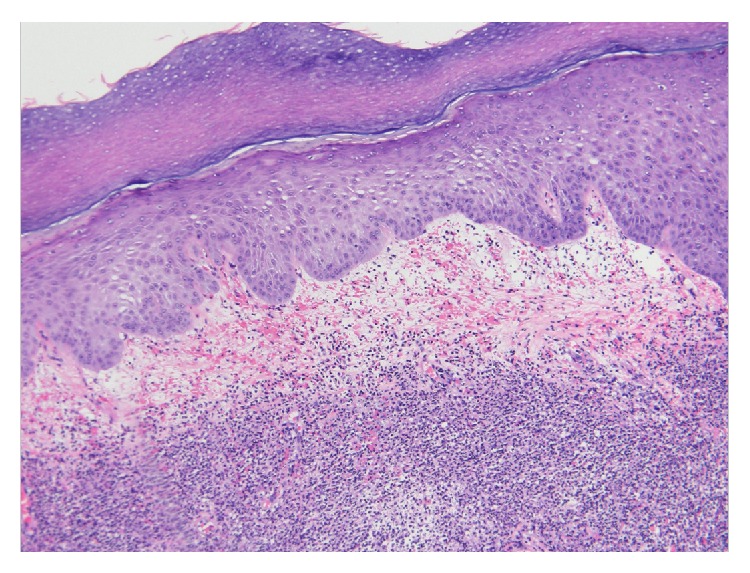
Histologic sections show acanthotic epidermis with marked papillary dermal edema with areas of hemorrhage. Also present is a dense band like neutrophilic infiltrate that extends from the superficial to middermis.

A 54-year-old male presented to our clinic with a four-year history of SS with ulcers on his fingers along with numbness and paresthesias of the extremities and rapidly progressive hearing loss. The patient was diagnosed with SS four years ago by another clinic after developing skin lesions involving all four extremities. He did not recall having any neurological symptoms at that time. A skin biopsy demonstrated neutrophilic infiltrate in the dermis with papillary dermal edema and no evidence of vasculitis (Figure 1). Special stains for infections were negative. These findings clinically correlated with a diagnosis of SS. The patient was started on prednisone at a dose of 20 mg daily and intralesional triamcinolone was used periodically to control active lesions. Despite the use of topical dapsone and silver sulfadiazine to the lesions at that time, he did require an amputation of a finger secondary to an ulcer and infection.

Over the next several months, the patient developed newer cutaneous lesions along with progressive numbness and paresthesias in the feet that would later also affect his hands. A neurological evaluation revealed that pinprick, vibratory, and position senses of the distal lower extremities were all decreased. Nerve conduction and needle EMG studies demonstrated severe sensorimotor peripheral neuropathy in all extremities with mixed axonal and demyelination features. The differential diagnosis included diabetic, nutritional, inflammatory, or toxic neuropathy.

Extensive laboratory studies done at this time were significant for an elevated white blood cell count of 13.8 (normal < 11 per microliter) with 90% neutrophils, mild macrocytic anemia (hemoglobin 13 g/dL, MCV 131), and low TSH of 0.254 (normal range: 0.35–5.5 international units/mL). Additionally, serum immunofixation electrophoresis demonstrated a monoclonal IgG kappa spike. Arsenic, lead, mercury, and cadmium levels were all within normal limits. The patient's hemoglobin A1C, vitamin B_12_, and ANA titer were within normal limits and rheumatoid factor was negative. An extensive paraneoplastic panel was also negative, including acetylcholine receptor antibody, striate muscle antibody, anti-Hu, anti-Ri, neuronal (V-G) potassium channel antibody, P/Q- and N-type calcium channel antibodies, anti-glial nuclear antibody, Purkinje cell cytoplasmic antibodies (types 1, 2, and Tr), amphiphysin antibody, and CRMP-5-IgG antibodies.

The patient subsequently underwent sural nerve biopsy that revealed a moderately severe axonal neuropathy with scattered digested myelin chambers and an estimated 40% loss of myelinated fibers. Cerebrospinal fluid analysis showed elevated protein of 177 mg/dL (normal: 15–45 mg/dL), red blood cell count of 12 (normal: 0-1/mm^3^), albumin of 141 mg/dL (normal: 13.9–24.6 mg/dL), and IgG of 12.6 mg/dL (normal: < 5.9). Glucose, white blood cell count, and myelin basic protein values were all within normal limits.

**Figure 2:**
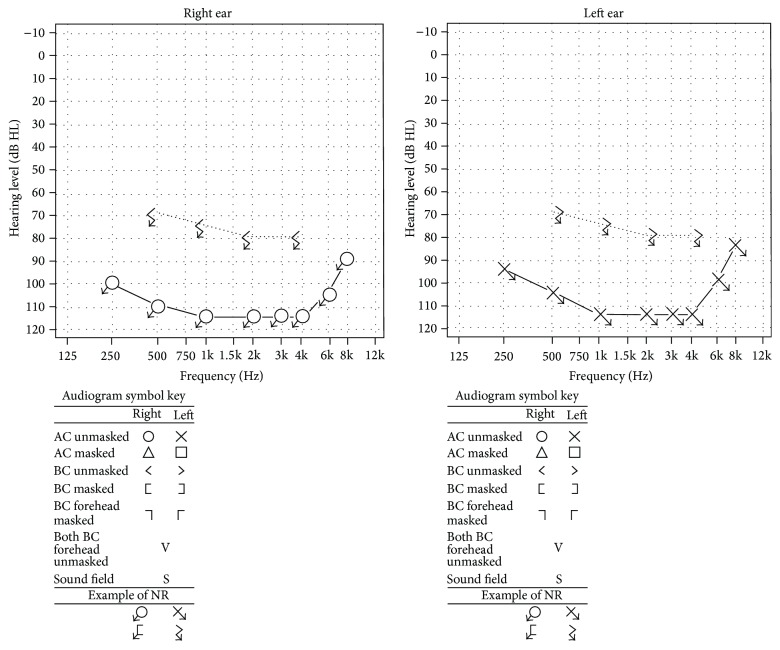
Bilateral severe SNHL was seen on the pure tone audiogram (precochlear transplantation).

The patient was concurrently evaluated for rapidly progressive hearing loss and associated dizziness with changes of head position and bilateral tinnitus. A trial of hearing aids the prior year had failed. An audiogram revealed profound sensorineural hearing loss bilaterally, with a word recognition score of 0% (Figure 2). There was no evidence of acoustic neuromas or any other abnormality on magnetic resonance imaging.

Hence, SS was considered to be the most likely underlying etiology of the patient's persistent nonhealing wounds on his extremities, peripheral polyneuropathy, and bilateral sensorineural deafness. The patient's cutaneous lesions did respond to azathioprine and intravenous immunoglobulin (IVIg). He also noticed a partial symptomatic relief of his neuropathy with the addition of IVIg. However, no beneficial response was observed for his hearing loss. The patient did not notice any further improvement with the addition of other systemic agents including mycophenolate mofetil, cyclosporine, pregabalin, gabapentin, and nortriptyline. The patient underwent bilateral cochlear implants from which he and his family have reported a subjective partial recovery of his hearing loss along with resolution of his vertigo and tinnitus. The patient has not noticed any subjective postoperative deterioration in his hearing nor relapse of other otological symptoms. However, he is due to undergo objective speech perception testing in two months.

## 3. Discussion

Sweet's syndrome is characterized by the acute onset of erythematous, tender nodules, or plaques of the skin that are composed of dermal neutrophilic infiltrate [2, 3]. A fever and abnormal laboratory values including leukocytosis with greater than 70% neutrophils, elevated erythrocyte sedimentation rate, and elevated C-reactive protein commonly accompany the cutaneous findings [2, 3]. While the cause of Sweet's syndrome is unknown, it seems to be reactive inflammation resulting in local or systemic cytokine secretion and immune dysregulation. This syndrome has been associated with a medication, infection, or underlying systemic etiology such as an autoimmune disorder or malignancy including plasma cell dyscrasias [2]. Systemic involvement has been also reported in association with the cutaneous presentation [2].

Neurologic manifestations of Sweet's syndrome are rare, with encephalitis and meningitis being the most common complications when they do occur. The majority of patients with neurologic symptoms improved when treated with corticosteroids. However, those with peripheral neuropathy have been reported to experience permanent sequelae [3].

There are two case reports in the English literature of bilateral sensorineural hearing loss patients with a history of Sweet's syndrome [4, 5]. While the pathogenesis of SS is not clear, its association with certain diseases including rheumatoid arthritis and relapsing polychondritis and response to systemic steroids has led to the hypothesis of a possible autoimmune pathogenesis. Successful treatment of autoimmune hearing loss with systemic steroids and cyclophosphamide was first reported in 1979 [1]. However, treatment with oral corticosteroids and plasmapheresis was able to only temporarily arrest the progression of the hearing loss in only one previously reported SS patient [4]. Both patients eventually had progressive hearing loss similar to our patient despite the usage of systemic steroids and multiple steroid sparing agents including mycophenolate mofetil, azathioprine, and methotrexate [4, 5]. This anecdotal experience would indicate that conventional immune suppression will not provide any long term benefit for the prevention of SNHL in SS. However, cochlear implantation may prove to be more beneficial as improvement in speech perception was observed in both our patient and one previously reported patient [5].

Our patient's SS was most likely secondary to the monoclonal gammopathy. All other possible etiologies of SS were tested and excluded. In previous reported cases including those with hearing loss, it has been observed that the association of SS with neurological and otological symptoms can result in irreversible sequelae. To our knowledge, there are no reported cases of both sensorineural hearing loss and peripheral neuropathy in a patient with associated Sweet's syndrome. Although the exact pathogenesis of all the above conditions is unknown, we suspect a common underlying immune dysregulation. This case highlights the need to recognize Sweet's syndrome as a complicated disease process where the role of otolaryngologists is important in the multidisciplinary coordination of care in both diagnosis and treatment. Further studies and research are needed to better understand the pathophysiology of SS with respect to hearing loss and neurological involvement.
